# Detection and molecular characterization of *Actinomyces denticolens* causing lymph node abscessation in horses

**DOI:** 10.3389/fvets.2023.1225528

**Published:** 2023-07-20

**Authors:** L. van den Wollenberg, C. van Maanen, R. Buter, P. Janszen, F. Rey, E. van Engelen

**Affiliations:** ^1^Royal GD (Animal Health Service), Deventer, Netherlands; ^2^Equine Clinic De Raaphorst, Wassenaar, Netherlands; ^3^Veterinary Clinic Winsum, Equine Division, Winsum, Netherlands

**Keywords:** horse, *Actinomyces denticolens*, lymph node abscess, oral associated infection, whole genome sequencing, draft genome

## Abstract

**Introduction:**

Abscessation of equine head lymph nodes can be caused by various bacteria, but Streptococcus *equi* subsp. *equi* is mainly involved. At our laboratory, samples of three unrelated horses with submandibular abscesses were found negative for *S. equi*, and further testing proved the presence of another genus. This raised the question for the exact identity of this pathogen and whether these isolates were epidemiologically related and it warranted further characterization with regards of virulence and resistance factors.

**Methods:**

Culture followed by identification using MALDI-TOF MS, MIC testing and whole genome sequencing (WGS) was performed to characterize the bacteria.

**Results:**

Bacterial culture and subsequent identification with MALDI-TOF MS resulted in the reliable identification of *A. denticolens* in two of the three cases. Final confirmation of *A. denticolens* for all three isolates was achieved by analysis of the WGS data, supported by multilocus sequence typing (MLST). The three isolates showed 95% nucleotide sequence identity. The number of single nucleotide polymorphisms (10,170 to 36,058) indicated that the isolates were not clonal, suggesting that these cases were epidemiologically unrelated. Only four known virulence related genes were detected. The absence of known antibiotic resistance genes was in line with the high susceptibility, as indicated by the susceptibility patterns obtained for two of the three isolates.

**Conclusion:**

We conclude that *A. denticolens* should be included in the differential diagnosis of (submandibular) lymph node abscessation in horses, especially if strangles cannot be confirmed with laboratory diagnostics. Furthermore, we report the first draft genome of *A. denticolens* isolated from horses.

## 1. Introduction

Many different bacteria can play a role in cervicofacial and upper respiratory tract infections in horses. In this respect, strangles is an important disease for horses and their owners. This highly contagious disease is caused by the bacterium *Streptococcus equi* subspecies *equi* (*S. equi*), which, in addition to respiratory symptoms, quite often causes lymph node abscesses. However, occasionally other bacteria are found to cause abscessation in the cervicofacial region. For example, *Actinomyces denticolens*, a species that has been sporadically described in recent years as the causative agent in case reports of such chronic inflammations (1–4). However, to date, these reports are scarce, and in horses, actinomycosis is considered to be a rare condition (4, 5).

*Actinomyces* spp. are gram-positive, non-spore-forming, facultative anaerobic rods that are part of the normal microbiota of the oral cavity and respiratory tract of humans and several animal species, including horses (6–8). They are recognized as possible causative agents of orally associated infections. As opportunistic pathogens, they incidentally invade the tissue and cause infection when mucosal barriers are disrupted (6, 9–11). In cattle, for example, *Actinomyces bovis* is typically introduced from the oral cavity into the gingiva and jawbone via mucosal injuries, usually as a result of dental disease or penetrating/migrating foreign bodies. The most common resulting clinical picture is mandibular osteomyelitis, known as “lumpy jaw syndrome” (12–14). “Lumpy jaw syndrome” is also the most common clinical manifestation seen in human actinomycosis, following a mucosal lesion due to, for example, gingivitis, dental extraction, or neoplastic conditions. The *Actinomyces* species predominantly found in this context are *A. israelii* and *A. gerencseriae* (7, 8).

*A. denticolens* was described for the first time in 1984 after isolation from dental plaque in healthy cattle (15). Over time, it was also isolated from the oral cavity and lower respiratory tract of healthy horses and donkeys (16). Recently, a study in slaughter horses identified *A. denticolens* as part of the common oral flora that colonizes the equine tonsil crypts (17).

Clinical signs in horses due to an infection with *A. denticolens* can be both local and generalized. These may include fever, anorexia, depression, painful swelling, and abscessation in lymph nodes or other soft tissues, followed by rupture and drainage of purulent material (1–4). In the cases reported so far, the infection was mainly localized in the submandibular lymph nodes. Taken together, this may result in a clinical picture very similar to that of strangles.

Laboratory confirmation of *A. denticolens* may be difficult because of a general lack of familiarity with this pathogen and a low success rate in culturing as a result of its slow-growing and fastidious nature (7). Furthermore, identification on the species level is demanding for a routine diagnostic laboratory because it requires additional phenotypic or genotypic diagnostic technologies such as MALDI-TOF MS or 16SrRNA sequencing (3, 7, 17–19). Communication between a clinician and microbiologist is of added value in this respect because the chances of laboratory confirmation may significantly improve when a targeted search for *A*. *denticolens* is carried out (8).

In this study, we report the identification and whole genome sequencing of three *A. denticolens* isolates originating from submandibular abscesses of unrelated horses. In addition, high genetic variability between the different isolates and the low presence of known virulence and antibiotic resistance genes were demonstrated.

## 2. Materials and methods

### 2.1. Animals

The three horses involved were privately owned and were presented to their respective veterinarians for similar clinical complaints: a swelling in the submandibular region. None of them had shown fever or nasal discharge during the time before, and they had no history of trauma, dental problems, or other problems in the head and neck region, except for Case 3. All three horses were single cases on their premises. After diagnosing abscessation of the submandibular lymph node, purulent material from each case was obtained and submitted to the laboratory of Royal GD (an organization that deals with animal health expertise, research and development, and diagnostics). With this material, *S. equi* real-time PCR, bacteriological culture, and subsequent antibiotic susceptibility tests were performed as described below. Furthermore, serum samples were submitted to test for the presence of antibodies specific to *S. equi*.

Case 1 concerned a 4-year-old Dutch warmblood gelding in which a submandibular mass had developed during the 4 weeks prior to presentation. The mass was 7 × 7 cm in size and not painful on palpation. The horse displayed no other clinical signs. An oral dental inspection showed no abnormalities. Dental X-rays of the affected area showed some widening of the alveolar space at 310 (lower left second molar) and 311 (lower left third molar), but no other abnormalities were apparent. Ultrasonographic examination revealed abscessation of the left mandibular lymph node with a thickened surrounding capsule without fistulation or communication with dental roots. After sampling, the abscess ruptured spontaneously. Subsequently, it was curetted and flushed twice daily with betadine diluted in sterile saline (0.9% NaCl). The wound was fully closed 6 weeks later, and a small amount of non-painful fibrous scar tissue remained. An inquiry conducted after 2 years revealed that no relapse had occurred since.

In Case 2, a 7-year-old Arabian mare was presented for evaluation of a recently discovered submandibular mass. The mass was 4 × 4 cm in size, painful on palpation, and surrounded by edema. Ultrasonographic examination revealed abscessation of the left mandibular lymph node without fistulation or communication with dental roots. The abscess ruptured spontaneously after 4 days, and some white-gray pus drained out of it; thereafter, it was opened further to aid drainage. After flushing with a diluted iodine solution for 4 more days, the abscess gradually closed, after which a small amount of fibrous scar tissue remained. After 18 months, no relapse had occurred.

**Figure 1:**
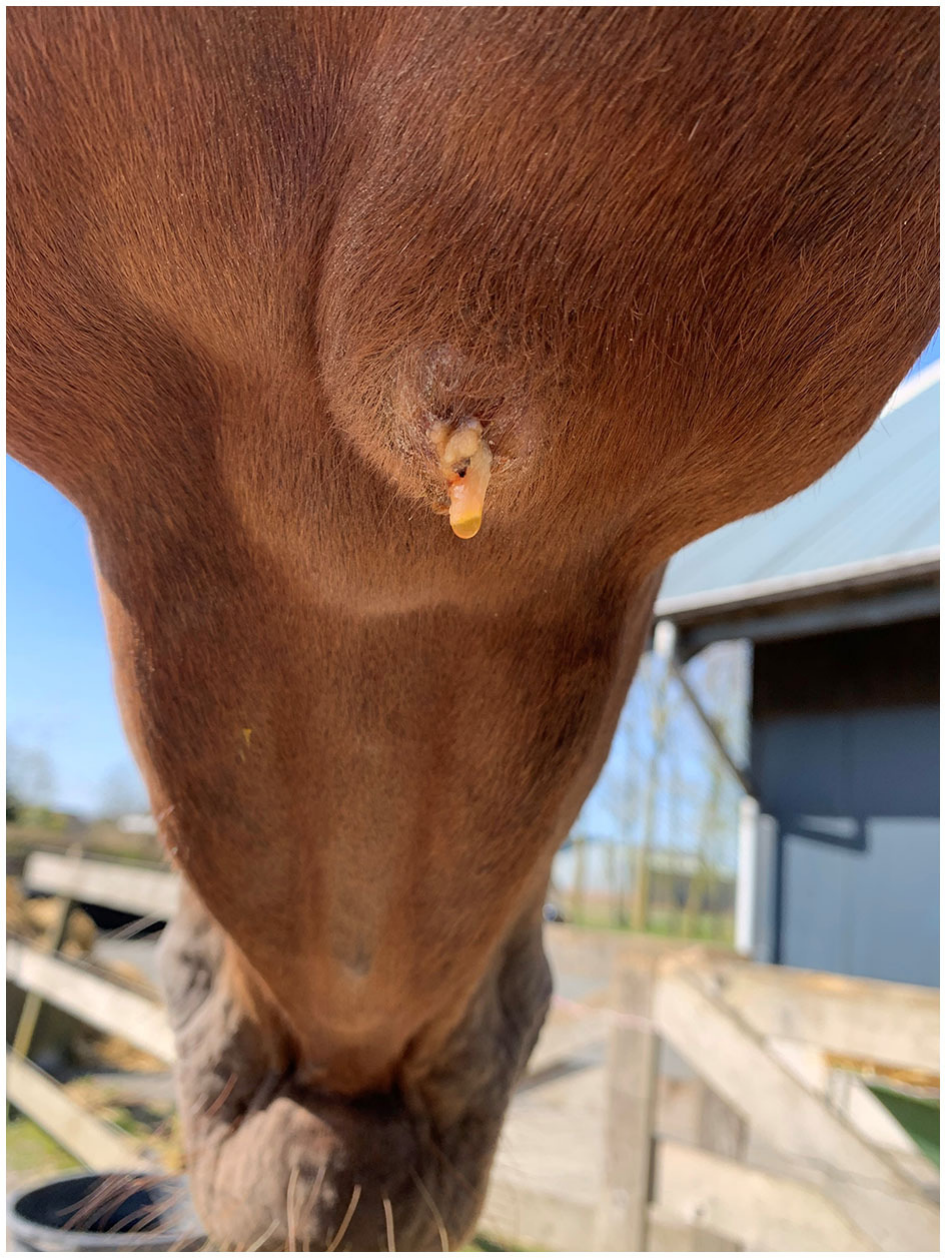
The right mandibular lymph node of the horse in Case 3 a few days after initial presentation.

Case 3 concerned a 5-year-old Dutch warmblood mare presented with a submandibular swelling that had developed recently. A week before, the veterinarian had been consulted because the horse had damaged the buccal mucosa on the left side while chewing willow branches. The submandibular mass was identified as being the right submandibular lymph node and was ~4 × 5 cm in size. After a few days, some purulent discharge draining out of it was noticed, as shown in Figure 1. In the following weeks, swelling and discharge decreased gradually; no fever was detected during this period. After 3 months, the lymph node suddenly increased considerably in size. At that point, a sample of purulent material from the lymph node was collected by means of aseptic aspiration, and the abscess was surgically opened. After opening, a large amount of gray-yellow pus was released. Subsequently, the abscess was flushed two times daily with a diluted iodine solution until closure after 5 days. The horse displayed no further clinical symptoms until 6 months later when the lymph node became slightly enlarged again (1.5 × 2 cm) in the absence of other clinical signs. When it was reopened, ~2 ml of purulent material came out, and it was flushed again. After 18 months, no more clinical signs had been noticed, and only a small fibrous nodule remained.

### 2.2. Laboratory diagnostics

#### 2.2.1. PCR

In Case 2 and Case 3, a nasal swab and a lymph node aspirate sample, respectively, were examined by real-time PCR specifically for *S. equi* using primers and probes targeting the *SeeI* gene (20). Samples with Ct ≤ 40 were regarded as positive for *S. equi*. Samples with Ct > 40 or without signal were regarded as negative for *S. equi*.

#### 2.2.2. Serological examination

In all three cases, a serum sample was taken more than 3 weeks after the onset of the first clinical symptoms and submitted for serological testing specifically for *S. equi* to exclude the possibility of strangles. For this purpose, an enhanced indirect ELISA test based on specific recombinant antigens A and C (Strangles ELISA kit, Animal Health Trust, Newmarket, UK) (21) was used in conformity with the recommendations of the manufacturer.

#### 2.2.3. Bacteriological examination

Specimens were cultured for bacterial growth by inoculating two sheep blood agar plates (Beef extract 10.0 g/L; Peptone 10.0 g/L; Sodium chloride 5.0 g/L; Agar 12.0 g/L; and sheep blood 60.0 ml/L, Biotrading, Moordrecht, the Netherlands) with a swab and subsequently spreading with a sequential dilution streaking pattern. One sheep blood agar plate was incubated aerobically and the other anaerobically for 48 h at 37°C. For anaerobic culture, the plates were placed in a jar, and the gas composition was changed in three cycles to a final composition of 5% H_2_, 9.9% CO_2_, 84.9% N_2_, and 0.0% (0.2%) O_2_ using a gas exchange device (Anoxomat, Advanced Instruments, USA). After culture, small white colonies were seen, which appeared to be firm and pitting the agar. These colonies were picked for phenotypic identification using MALDI-TOF MS (Sirius GP system, Bruker, Germany), using the compass for the FlexSeries 1.4 software, Flex Control version 3.4, and Compass Library DB-8468. This was done in duplicate without and with additional sample treatment with formic acid. In general, a score value higher than 2.0 was regarded as confident identification on the species level, between 1.7 and 2.0 as confident identification on the genus level, and below 1.7 as unreliable identification.

#### 2.2.4. MIC testing

Antimicrobial susceptibility was determined using the broth microdilution method for bacteria isolated from animals, as described in the document VET01-A4 from the Clinical and Laboratory Standards Institute (https://clsi.org/media/1531/vet01a4_sample.pdf). For this bacterium, cation-adjusted Mueller-Hinton broth (CAMHB; Becton, Dickinson and Company, Vianen, the Netherlands) with lysed horse blood was used. Routinely used customized microtiter plates (Merlin Diagnostics, Bornheim-Hersel, Germany) were used at the Bacteriology Laboratory of Royal GD Animal Health. Plates were incubated aerobically at 35°C for 22 h and an additional 24 h if growth was not sufficient. Minimal inhibitory concentration (MIC) values were determined by visual reading of the plates and comparing growth at different concentrations of antibiotics with growth control.

#### 2.2.5. Whole genome sequencing

For further identification, all three strains were analyzed by whole genome sequencing (WGS). The extracted DNA was used for the preparation of libraries using the Nextera XT DNA library preparation kit (Illumina, San Diego, CA, USA). 300-cycle sequence reads (2 × 150 bp paired-end) were generated using the Illumina NovaSeq 6000 system. The initial quality assessment was based on data passing the Illumina chastity filtering. Subsequently, reads containing PhiX control signals were removed using an in-house filtering protocol (Baseclear, Leiden, the Netherlands). In addition, reads containing (partial) adapters were clipped (up to a minimum read length of 50 bp). The second quality assessment was based on the remaining reads using the FASTQC quality control tool version 0.11.5. De-multiplexed and adapter-clipped reads were analyzed using the CLC Genomics Workbench version 21.0.4 (Qiagen Aarhus A/S, Aarhus, Denmark).

*De novo* assembly was performed using the slow mapping mode with a minimum expected contig size of 1,000 nucleotides. The maximum paired read distance allowed was 1,000 nucleotides. The parameters for mapping back the reads were mismatch cost = 2; insertion cost = 3; deletion cost = 3; length fraction = 0.5; and similarity fraction = 0.8.

Reference assembly was performed using the genome of *A. denticolens* strain Chiba 101 (GenBank: NZ_AP017896), isolated from an arthritic leg joint of a pig raised in Japan (22) as a reference sequence. The parameters for mapping the reads were as follows: match score = 1; mismatch cost = 2; insertion open cost = 6; insertion extend cost = 1; deletion open cost = 6; deletion extend cost = 1; length fraction = 0.5; and similarity fraction = 0.8. Local realignment was performed using the following parameters: unaligned ends were realigned; multi-pass realignment = 2. The raw sequencing reads were submitted to the SRA database by NCBI and registered under the following accession numbers: Case 1: gdb-43843, SRR16962209; Case 2: gdb-38104, SRR16962208; and Case 3: gdb-38528, SRR16962207.

Variant detection was performed using the Basic Variant Detection script version 2.1 with the following parameters: ploidy = 2; ignore positions with coverage above 5,000; ignore broken pairs; minimum coverage = 20; minimum count = 15; minimum frequency = 35%; include basic quality filtering; neighborhood radius = 5; minimum central quality = 20; and minimum neighborhood quality = 15. From the resulting variant tracks, a radial unrooted phylogenetic tree with neighbor-joining clustering was created to demonstrate genetic diversity. The parameters for the construction of the SNP tree were as follows: MNVs not included, the minimum coverage required in each sample = 20, minimum coverage percentage of the average required = 20, prune distance = 0, and minimum *z*-score required = 0.0. In addition to the NGS data from the three *A. denticolens* strains obtained in this study, for two strains, NGS data were included from the Sequence Read Archive (SRA), an NCBI-maintained database of NGS data. These were strain DSM 20671, isolated from the dental plaque of cattle, and strain PA, isolated from the rumen of ruminant livestock. These SRA reads were deposited in the SRA database under accession numbers DRR072224 and SRR4228336, respectively. Since very limited whole genome sequences of *A. denticolens* strains were available, sequences from these two confirmed *A. denticolens* strains were added to the variant detection analysis.

Based on the detected variants, an SNP matrix was constructed for the three *A. denticolens* isolates in this study and the strains from the SRA database. Following reference assembly from the read mappings, a whole genome consensus sequence was constructed, which was used to perform a K-mer tree using the Create K-mer tree script, version 1.3, and the following parameters were used: K-mer length = 16 bp and only index k-mers with prefix = ATGAC (Method = FFP and Strand = Both strands). Besides the three strains from this study, the whole genome sequences from 21 *Actinomyces* spp. strains deposited in Genbank were also included in the K-mer tree.

The contigs from the *de novo* assembly were used for the identification of acquired antimicrobial resistance genes using the Find Resistance with Nucleotide DB script version 1.2 and the ResFinder Database (version 2021-02-28, minimum identity % = 75, minimum length % = 60%, and filter overlaps = Yes). For the identification of genes that may contribute to virulence, the same tool and settings were used but in combination with the Virulence Factor Database (VFDB, full dataset; version 2021-08-24). Finally, the sequence reads were mapped against a plasmid database (Plasmid database, NCBI, 2020-10-05) using the Taxonomic Profiling script version 2.33 (settings: filter host reads = no, auto-detect paired distances = Yes, abundance table = Yes, minimum seed length = 30, adjust read count abundances = Yes, unclassified reads = Yes, and report = Yes) in order to find plasmids that might be related to resistance or virulence using the NCBI plasmid database (version 2020-10-05).

Since only limited whole genome sequences for *A. denticolens* were available, genotyping using multilocus sequence typing (MLST) was also performed. For MLST analysis, an MLST scheme using seven housekeeping genes (*atpA, gltA, gyrA, metG, pgi, pheS*, and *rpoB*) was used as developed by Henssge et al. (23) for genotyping of *A. oris* and *A. naeslundii* isolates. In our study, this MLST scheme was extended with allelic profiles and sequence types (STs) for the *A. denticolens* strains described previously and the isolates from this study, as well as for eight other *Actinomyces* species. For this purpose, the sequences of the seven housekeeping genes were extracted from the whole genome sequences of these strains that were downloaded from Genbank and that were also used for the construction of the K-mer tree. The DNA fragments from *A. oris* strain A18A-3 (respectively GenBank EU620779, EU620895, EU621011, EU621127, EU603149, GQ354616, and EU621243) were used as templates. Accession numbers for the partial housekeeping gene sequences from *A. oris* and *A. naeslundii* isolates and sequences from the other nine *Actinomyces* strains were previously reported by Henssge et al. (23) and were downloaded from Genbank. Together with the sequences from the three isolates from this study, they were imported in BioNumerics Bioinformatics Package e v 7.6 (Applied Maths, Sint-Martens-Latem, Belgium) together with the corresponding allelic profiles and ST. For each housekeeping gene, phylogenetic analysis was performed using the multiple alignment method followed by single linkage clustering, and new allelic profiles were identified. Continuous new numbers were assigned to these new allelic profiles. Concatenated sequences from the seven MLST genes were constructed, and also from these sequences, a multiple alignment was performed, and a phylogenetic tree was constructed using the single linkage method. Bootstrap values were calculated based on 1,000 recalculations of the tree. Based on separated branches, new STs were identified, also by continuous numbering from the existing ST for *A. oris* and *A. naeslundii*. Finally, a maximum parsimony tree was constructed from all concatenated sequences in order to get better insight into the genetic relatedness between the *A. denticolens* isolated from this study and the two *A. denticolens strains* Chiba 101 (Genbank AP017896) and NCTC 11490 (Genbank NZ_UFSA00000000.1), but also with other *Actinomyces* species. The tree was drawn on a logarithmic scale. The MLST sequences for the three *A. denticolens* isolates in this study were submitted to Genbank and registered under accession numbers OM489450 to OM489470.

## 3. Results

### 3.1. Diagnostics applied for the detection of *S. equi*

Real-time PCR, culture, and serology specific to *S. equi* yielded negative results in all samples submitted for the three cases.

### 3.2. Culture and identification with MALDI TOF

After 48 h culture, numerous small white colonies were seen, which appeared to be firm and pitting the agar. In two cases, this was a pure culture; in one case, there were a few other colonies present besides the rich growth of the small white colonies. When the colonies of the isolates in the three cases were tested using MALDI-TOF MS, the overall score values of the first hits varied between 1.15 and 2.03. From 12 measurements (four per isolate), nine times, *A. denticolens* was the first hit. When *A. denticolens* was the first hit, the score value varied between 1.60 and 2.03. The score values of the second hits were always below 1.38. For Case 1, with the use of formic acid, *A. denticolens* could be identified with a score of 1.88–2.03, but without formic acid, *A. denticolens* was found one time with a score value of 1.82, and another sample yielded the identification of another bacterium with a very low confidence score (1.15). For Case 2, no reliable identification could be given. Maximum scores were found with the use of formic acid for *A. denticolens*, however, with score values between 1.60 and 1.69, which is inconclusive.

For Case 3, both without and with formic acid, the bacterium was identified as *A. denticolens*, with higher confidence scores without formic acid (1.98 and 1.90) compared to those with formic acid (1.77 and 1.86).

Overall, the identification of *A. denticolens* with MALDI-TOF was highly reliable for Case 1 and sufficiently reliable for Case 3, but for Case 2, the results remained inconclusive.

### 3.3. MIC testing

MIC values could be determined for Case 1 and Case 3, but not for Case 2, because for this isolate, growth in the medium used for MIC was not sufficient. With the use of antibiotic concentrations in our standard panel, Case 1 and Case 3 showed the same pattern of susceptibility. For enrofloxacin, MIC values of 2 or 4 μg/ml were found, which was regarded as presumed resistant, and for trimethoprim/sulfamethoxazole, MIC values of 0.0615/1.187 and 0.25/4.75 μg/ml were found, which was regarded as presumed sensitive. For the other antibiotics, there was no growth at any concentration tested, which was regarded as presumed sensitive. Results are shown in Table 1.

**Table 1:** MIC values of the *A. denticolens* isolates using the microbroth dilution assay.

	**Case 1**	**Case 3**
Antibiotic	MIC value (μg/ml)	MIC value (μg/ml)
Amoxicillin/Clavulanic acid	≤ 0.25/0.125	≤ 0.25/0.125
Ampicillin	≤ 0.0625	≤ 0.0625
Cefepine	≤ 0.5	≤ 0.5
Ceftiofur	≤ 0.25	≤ 0.25
Clindamycin	≤ 0.25	≤ 0.25
Enrofloxacin	2	4
Erythromycin	≤ 0.125	≤ 0.125
Florfenicol	≤ 2	≤ 2
Neomycin	≤ 4	≤ 4
Oxacillin	≤ 0.25	≤ 0.25
Penicillin G	≤ 0./0625	≤ 0.0625
Sulfamethoxazole	≤ 16	≤ 16
Trimethoprim/Sulfamethoxazole	0.0615/1.187	0.25/4.75
Tetracycline	≤ 0.25	≤ 0.25
Tilmicosin	≤ 4	≤ 4

Values are given for Case 1 and Case 3. In Case 2, the growth was not sufficient for MIC testing.

### 3.4. WGS and MLST

The *de novo* assembly of the sequencing data of *A. denticolens* isolated from Case 1, Case 2, and Case 3 resulted in 159, 163, and 118 contigs, respectively. For all three isolates, the sum of all contigs sizes was ~2.7 to 2.8 Mb. Reference coverages of 92.6, 91.4, and 89.7% were found for the isolates from Case 1, Case 2, and Case 3, respectively, using strain Chiba 101 (NZ_AP017896) as the reference strain. The average genome coverage was 264, 285, and 471, respectively. The good coverage, together with the fact that the size of the complete genome from *A. denticolens* reference strain Chiba 101 (NZ_AP017896) was very similar (2.877.864 bp), indicated that the genomes of the three *A. denticolens* isolates in this study were relatively complete and were assembled with good reliability.

**Figure 2:**
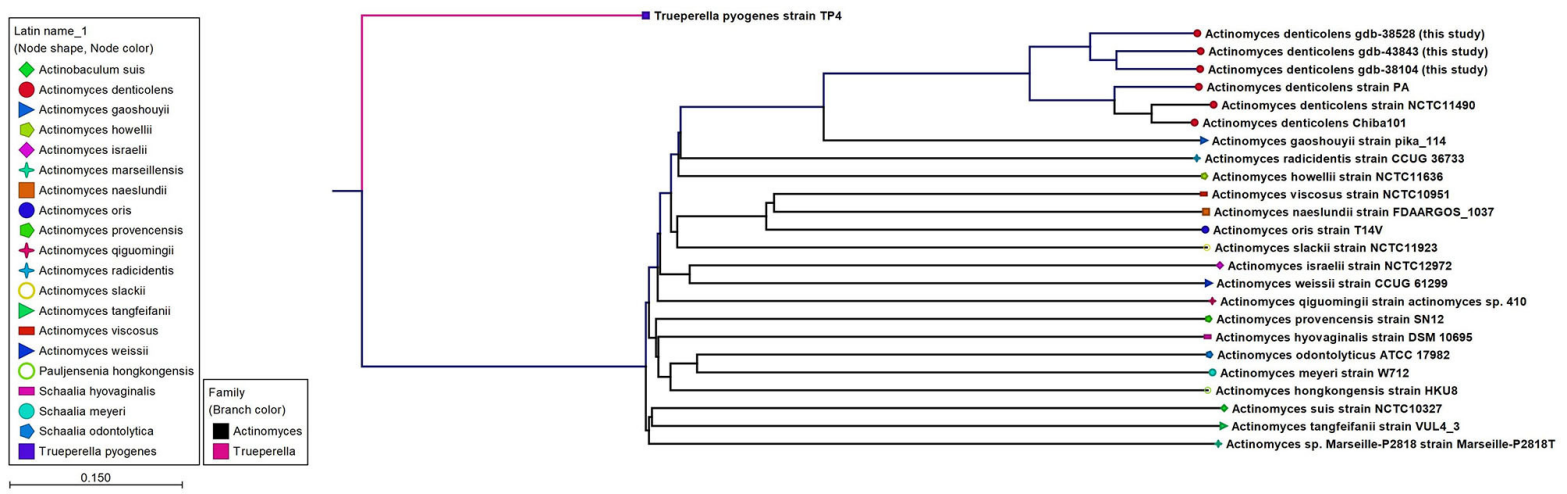
An unrooted K-mer tree constructed by the neighbor joining method using the distance function Feature Frequency Profile via Jensen-Shannon divergences (FFP). The tree is constructed from 16-mers with the prefix ATGAC extracted from consensus read mappings of *A. denticolens* isolates GDB-43843, GDB-38528, GDB-38104, and strain PA. Additionally, whole genome nucleotide sequences from *A. denticolens* strain Chiba101, isolated from an arthritic leg joint of a pig raised in Japan (Genbank: AP017896), and strain NCTC11490 (also named ATCC 43322; DSM 20671; SN8/4303, Genbank NZ_UFSA00000000.1, isolated from cow dental plaque), as well as whole genome nucleotide sequences from 19 other *Actinomyces* species downloaded from Genbank, were added to the tree. Nodes are colorized and symbolized on the species level, and included species are also mentioned next to the nodes.

WGS results confirmed the MALDI-TOF identification of *A. denticolens* for Case 1 and Case 3 and completed the identification of *A*. *denticolens* for Case 2. Identification results with the MALDI-TOF were in concordance with the K-mer tree shown in Figure 2, which was constructed from the consensus sequences extracted from the read mappings.

In total, 53,686 single nucleotide polymorphisms (SNPs) were informative and used for the construction of the SNP tree, as shown in Table 2.

**Table 2:** SNP matrix containing the pairwise number of SNP differences between all pairs of isolates included in the analysis and based on 53,686 informative SNPs from five *A. denticolens* isolates using the *A. denticolens* Chiba 101 strain as a reference (NZ_AP017896).

**Sample name**	**DSM 20671 DRR072224**	**GDB-38104**	**GDB-38528**	**GDB-43843**	**Strain PA SRR4228336**
DSM 20671 DRR072224	0				
GDB-38104	35,681	0			
GDB-38528	33,503	20,139	0		
GDB-43843	34,995	15,785	19,339	0	
Strain PA SRR4228336	10,170	36,058	34,070	35,719	0

In addition to the identification results, the SNP matrix showed that there was neither a genetic relationship between the genome sequences of the *A. denticolens* isolates from the three cases reported here nor between the genome sequences of our isolates and the NCBI isolate PA. The minimum and maximum SNP differences among isolates were 10,170 and 36,058, respectively. MLST showed similar results; different sequence types (STs) were found for the three isolates. GDB-38104 was assigned to ST 110, GDB-38528 to ST 111, and GDB-43843 to ST 112. All three isolates were different for at least three alleles, indicating that they are unrelated. For strain Chiba 101 and strain NCTC 11490 (not included in the WG SNP tree), the same sequence type, ST 113, was found.

The whole genome sequences of the three isolates were investigated for the presence of acquired resistance genes and genes that may contribute to virulence. Despite the use of relatively low stringency criteria for minimum identity and minimum length, no genes with known relationships to resistance were found. In total, four genes associated with virulence were detected (*sigA/rpoV, rmlA, ahpC*, and *Rv0440*). Of these, the gene ahpC was only present in the isolate from Case 3.

## 4. Discussion

Within a relatively short period of time, we isolated, identified, and sequenced three *A. denticolens* isolates originating from submandibular abscesses of different, unrelated warmblood horses based in the Netherlands. Since 2008, only a limited number of reports have described the isolation of *A. denticolens* from abscesses in cervicofacial lymph nodes and soft tissues in horses (1–4). Therefore, equine veterinarians may be unaware of *A. denticolens* as a potential causative agent in such cases; logically, they will first think of strangles as a result of *S. equi* infection. In contrast to a typical case of upper respiratory tract infection caused by *S. equi*, fever is less commonly reported in horses with *A. denticolens* infection, and in addition, abscesses may develop more slowly. Furthermore, unlike the sequela seen in strangles, horses with *A. denticolens* infections do not appear to develop guttural pouch empyema and purulent nasal discharge (1–3). Despite these differences, *A. denticolens* should be in the differential diagnosis of cervicofacial abscessation due to *S. equi*, especially when it comes to cases that cannot be confirmed with laboratory diagnostics specific for S. *equi* such as PCR and serology (1–3, 17). The distinction is important since, in the case of strangles, stringent implications for biosecurity and management have to be considered for equine premises, while the identification of A. *denticolens* as the causative agent does not compel the need for these types of measures (3).

With regard to the etiology of *A. denticolens* infections in horses, mucosal lesions might be a “porte d”entrée,” just as is presumed for these types of infections in cattle and humans (7, 12, 14). One horse described in this report had indeed recently suffered a mucosal lesion in the oral cavity, although on the contralateral side of the abscess. However, no such prequel was known for the other two horses. Murakami et al. (17) stated that *A. denticolens* may colonize equine tonsils and may translocate to the lymph nodes of the cervicofacial region, similar to *S. equi*, and provoke infection, for example, in immunosuppressed horses (17). However, apart from the localized abscesses due to *A. denticolens* infections, these horses were healthy and in good condition, and there was no indication of immunosuppression in any of the horses included in our study. In general, the ultimate trigger of infection by this opportunistic pathogen cannot always be determined with certainty (1, 3, 4).

In our preliminary efforts to identify the bacterium, we utilized a MALDI-TOF MS system. Despite conducting repeated tests under varied conditions, we found that the identification of one strain at the species level was not sufficiently reliable. For identification, we used the on-target extraction method. Results might have been improved if the in-tube extraction method had been used. This aligns with a study conducted in 2014 (8), where the authors recognized the need for improvements in the use of MALDI-TOF for identifying *A. denticolens*. MALDI-TOF identification depends on the library used. In our laboratory, we use the manufacturer's standard library, which, at present, contains only one *A. denticolens* isolate. The consequence is that the identification of a phenotypically different *A. denticolens* is problematic. The ability to identify this bacterium with greater certainty will definitely improve when more different isolates are included in the library.

The definitive identification of *A. denticolens* as the causative bacterium was achieved in all three cases by means of WGS. The relationship to the *A. denticolens* type strain genomes in the K-mer tree confirmed the identity of these bacteria. WGS as a method for bacterial strain typing and epidemiologic analysis is primarily managed by SNP and/or gene by gene (e.g., core genome MLST) comparisons (24). Since for *Actinomyces denticolens*, no core genome MLST (cgMLST) scheme is available yet, SNP analysis from the core genome via reference-based mapping was performed. Whole genome SNP analyses from the isolates described in this study revealed that these isolates varied by 15,785 to 20,139 SNPs from each other. Genetic relatedness based on core genome sequences is still under discussion and is also organism-specific since the mutation rate can range widely between species (24). For *A. denticolens*, no species-specific cutoff is reported, but for other species, criteria for relatedness thresholds based on wg/cgMLST (allele) SNPs are suggested (24) to vary from ≤ 3 for *Acinetobacter baumannii* to ≤ 37 for *Pseudomonas aeruginosa*. Compared with the suggested SNPs, the number of SNPs in the SNP matrix from our *A. denticolens* isolates indicates that these isolates were not clonal, supporting the hypothesis that these cases are epidemiologically unrelated. Only a low number of virulence-associated genes were found; however, the number of virulence-associated genes is not necessarily correlated with virulence. As less research has been done on the virulence of *A. denticolens*, relevant genes might not be included in the virulence database (VFDB). Also, no known plasmids present in the used plasmid database were found in the WGS data from the three *A. denticolens* isolates. Therefore, the acquisition of plasmids harboring genes associated with virulence or resistance could not be confirmed.

**Figure 3:**
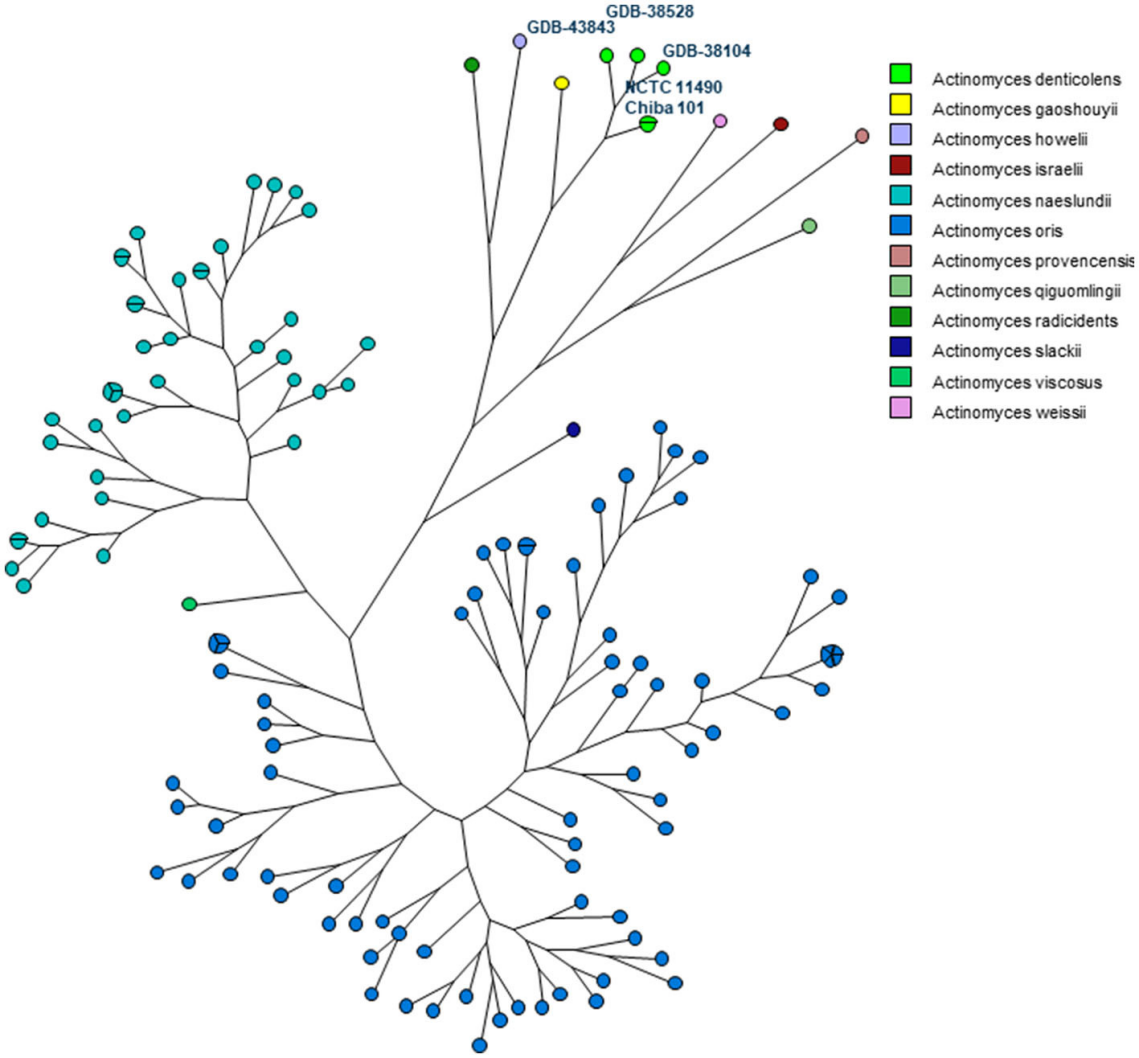
Maximum parsimony (MP) tree based on the concatenated sequences of seven housekeeping genes (atpA, gltA, gyrA, meG, pgi, pheS, and rpoB) present in 11 *Actinomyces* species. The nodes in the tree represent the isolates included. Small circles contain only one isolate, and bigger circles contain two or more isolates, as indicated by the parts of that circle. Each Actinomyces species is identified by a unique color. Nodes with the same color belong to the same species, as shown in the legend.

In addition, an MLST scheme developed for the identification and genotyping of *A. oris* and *A. naeslundii* (23) was used and extended for *A. denticolens* and eight other *Actinomyces* species. The housekeeping genes of this MLST scheme were previously used by Murakami et al. for comparing *A. denticolens* strain Chiba 101 with strain DSM20671 (5). However, they did not assign and report allelic profiles and STs for these strains. Therefore, to the best of our knowledge, this is the first time that an MLST scheme has been used for genotyping other *Actinomyces* species other than *A. oris* and *A. naeslundii*. Figure 3 shows the MP tree in which all *A. denticolens* isolates and strains form a distinct branch together with *A. gaoshouii* strain Pika 114. This strain appeared to be the most closely related *Actinomyces* species according to this MLST, which was also observed in the WGS K-mer tree. However, the latter method showed a clear separation between both species, which indicates that it has a higher discriminating power than MLST. The MLST results support the conclusion drawn from the WGS that the bacteria we isolated belong to the species *A. denticolens* but were unrelated.

MIC testing revealed that our *A. denticolens* isolates are highly susceptible to commonly used antibiotics. This fits with our observation that no known genes associated with antimicrobial resistance were present in the genome. Although both methods are not fully decisive, this corresponds with earlier descriptions of this genus. For example, a study based on 392 human clinical isolates found *Actinomyces* spp. to be almost uniformly susceptible to β-lactam antimicrobials, carbapenems, tetracyclines, and vancomycin (25). Since β-lactam antimicrobial agents have a high therapeutic index, in human medicine, they are considered to be the first choice of treatment (7, 8, 25).

In the equine cases reported in the literature so far, although limited in number, drainage and lavage of the abscess, in some cases, in combination with antimicrobial therapy, usually resulted in a rapid response with complete resolution of the abscess. However, the recurrence of abscesses and the necessity of prolonged treatment have also been reported (1–4). This corresponds to the three cases in this report, which are fairly localized and uncomplicated cases of abscessation. They were treated solely by draining and lavaging, and only one of them showed a relapse, limited in severity, after 6 months and was free of clinical signs after 18 months.

## 5. Conclusion

In this study, we describe the involvement of *A. denticolens* in equine submandibular lymph node abscess formation. WGS and MLST confirmed that the bacteria we isolated belong to the species *A. denticolens* but are not related. In addition, we report the first draft genome of *Actinomyces denticolens* isolated from horses.

## Data availability statement

The datasets presented in this study can be found in online repositories. The names of the repository/repositories and accession number(s) can be found at: https://www.ncbi.nlm.nih.gov/genbank/, SAMN23181213; https://www.ncbi.nlm.nih.gov/genbank/, SAMN23181212; https://www.ncbi.nlm.nih.gov/genbank/, SAMN23181211.

## Author contributions

PJ and FR collected the samples and provided the patient information. LW, EE, and RB organized for execution and interpretation of the diagnostic techniques. EE and RB analyzed the data. LW wrote the first draft of the manuscript. LW, EE, RB, PJ, FR, and CM wrote sections of the manuscript. All authors contributed to manuscript revision, read, and approved the submitted version.
